# Supplementary material for: DNA-based diversity assessment reveals a new coral barnacle, Cantellius alveoporae sp. nov. (Balanomorpha: Pyrgomatidae) exclusively associated with the high latitude coral Alveopora japonica in the waters of southern Korea
Source: PeerJ. 2021 Apr 29;9:e11284. doi: 10.7717/peerj.11284 (PMC8088765; doi:10.7717/peerj.11284)
Supplement: Table S1 [file peerj-09-11284-s001.docx]

**Table S1 Information on collection sites and Genbank no. for the sequences used for the phylogenetic tree.** —: not available.

| Voucher no. | Species name | Host | COI Reference | Accession no. COI | 12S Reference | Accession no. 12S |
| --- | --- | --- | --- | --- | --- | --- |
| UF8634 | *Cantellius* sp.1 | Philippines on coral  *Pachyseris rugosa* | Malay & Michonneau, 2014 | HG970542 | Malay & Michonneau, 2014 | HG970484 |
| UF8636 | *Cantellius* sp.1 | Philippines on coral  *Pachyseris rugosa* | Malay & Michonneau, 2014 | HG970543 | Malay & Michonneau, 2014 | HG970485 |
| CEL-KT35-3 | *Cantellius* sp.1 | Kenting Taiwan, on coral *Pachyseris peciosa* | Chan et al., 2018 | MG878745 | Tsang et al., 2014 | KF776150 |
| UF8670 | *Cantellius* sp.2 | Philippines on coral  *Montipora* sp. | Malay & Michonneau, 2014 | HG970552 | Malay & Michonneau, 2014 | HG970494 |
| UF8638 | *Cantellius* sp.3 | Philippines on coral *Porites* sp. | Malay & Michonneau, 2014 | HG970558 | Malay & Michonneau, 2014 | HG970500 |
| UF8663 | *Cantellius* sp.3 | Philippines on coral *Porites* sp. | Malay & Michonneau, 2014 | HG970551 | Malay & Michonneau, 2014 | HG970493 |
| UF8664 | *Cantellius* sp.4 | Philippines on coral  *Montipora* sp. | Malay & Michonneau, 2014 | HG970555 | Malay & Michonneau, 2014 | HG970497 |
| UF6541 | *Cantellius* sp.5 | Philippines on coral *Acropora* sp. | Malay & Michonneau, 2014 | HG970571 | Malay & Michonneau, 2014 | HG970510 |
| UF8676 | *Cantellius* sp.6 | Philippines on coral *Acropora* sp. | Malay & Michonneau, 2014 | HG970553 | Malay & Michonneau, 2014 | HG970495 |
| UF8651 | *Cantellius* sp.7 | Philippines on coral *Acropora* sp. | Malay & Michonneau, 2014 | HG970554 | Malay & Michonneau, 2014 | HG970496 |
| CEL-GI164-1 | *Cantellius*  *acutum* | Green Island, Taiwan on coral *Acropora lutkeni* | Chan et al., 2018 | MG878744 | Tsang et al., 2014 | KF776142 |
| acut1 | *Cantellius*  *acutum* | Unknown | — | — | Zweifler et al., 2020 | MG840382 |
| arc1 | *Cantellius arcuatum* | *Acropora* sp. | — | — | Zweifler et al., 2020 | MG840379. |
| CEL-KT101-3 | *Cantellius arcuatum* | Nanwan, Kenting, Taiwan on coral *Porites* sp. | — | — | Tsang et al., 2014 | KF776144 |
| CEL-KT26-3 | *Cantellius arcuatum* | Nanwan, Kenting, Taiwan on coral *Porites* sp | — | — | Tsang et al., 2014 | KF776143 |
| CEL-JJ01-02 | *Cantellius arcuatus* | Jeju Island, Korea on coral *Montipora millepora* | Chan et al., 2018 | MG878629 | Chan et al., 2018 | MG878752 |
| CEL-JJ01-03 | *Cantellius arcuatus* | Jeju Island, Korea on coral *Montipora millepora* | Chan et al., 2018 | MG878630 | Chan et al., 2018 | MG878753 |
| CEL-JJ01-04 | *Cantellius arcuatus* | Jeju Island, Korea on coral *Montipora millepora* | Chan et al., 2018 | MG878631 | Chan et al., 2018 | MG878754 |
| CEL-JJ02-02 | *Cantellius arcuatus* | Jeju Island, Korea on coral *Montipora millepora* | Chan et al., 2018 | MG878632 | Chan et al., 2018 | MG878755 |
| CEL-JJ02-03 | *Cantellius arcuatus* | Jeju Island, Korea on coral *Montipora millepora* | Chan et al., 2018 | MG878633 | Chan et al., 2018 | MG878756 |
| CEL-JJ02-04 | *Cantellius arcuatus* | Jeju Island, Korea on coral *Montipora millepora* | Chan et al., 2018 | MG878634 | Chan et al., 2018 | MG878757 |
| CEL-JJ13-02 | *Cantellius arcuatus* | Jeju Island, Korea on coral *Montipora millepora* | Chan et al., 2018 | MG878664 | Chan et al., 2018 | MG878779 |
| CEL-JJ13-03 | *Cantellius arcuatus* | Jeju Island, Korea on coral *Montipora millepora* | Chan et al., 2018 | MG878665 | Chan et al., 2018 | MG878780 |
| CEL-JJ13-05 | *Cantellius arcuatus* | Jeju Island, Korea on coral *Montipora millepora* | Chan et al., 2018 | MG878666 | Chan et al., 2018 | MG878781 |
| CEL-JJ14-02 | *Cantellius arcuatus* | Jeju Island, Korea on coral *Montipora millepora* | Chan et al., 2018 | MG878667 | Chan et al., 2018 | MG878782 |
| CEL-JJ14-03 | *Cantellius arcuatus* | Jeju Island, Korea on coral *Montipora millepora* | Chan et al., 2018 | MG878668 | Chan et al., 2018 | MG878783 |
| CEL-JJ14-04 | *Cantellius arcuatus* | Jeju Island, Korea on coral *Montipora millepora* | Chan et al., 2018 | MG878669 | Chan et al., 2018 | MG878784 |
| CEL-JJ15-01 | *Cantellius arcuatus* | Jeju Island, Korea on coral *Montipora millepora* | Chan et al., 2018 | MG878670 | Chan et al., 2018 | MG878785 |
| CEL-JJ15-02 | *Cantellius arcuatus* | Jeju Island, Korea on coral *Montipora millepora* | Chan et al., 2018 | MG878671 | Chan et al., 2018 | MG878786 |
| CEL-JJ15-05 | *Cantellius arcuatus* | Jeju Island, Korea on coral *Montipora millepora* | Chan et al., 2018 | MG878672 | Chan et al., 2018 | MG878787 |
| CEL-JJ16-02 | *Cantellius arcuatus* | Jeju Island, Korea on coral *Montipora millepora* | Chan et al., 2018 | MG878673 | Chan et al., 2018 | MG878788 |
| CEL-JJ16-03 | *Cantellius arcuatus* | Jeju Island, Korea on coral *Montipora millepora* | Chan et al., 2018 | MG878674 | Chan et al., 2018 | MG878789 |
| CEL-JJ16-04 | *Cantellius arcuatus* | Jeju Island, Korea on coral *Montipora millepora* | Chan et al., 2018 | MG878675 | Chan et al., 2018 | MG878790 |
| CEL-JJ17-02 | *Cantellius arcuatus* | Jeju Island, Korea on coral *Montipora millepora* | Chan et al., 2018 | MG878676 | Chan et al., 2018 | MG878791 |
| CEL-JJ17-03 | *Cantellius arcuatus* | Jeju Island, Korea on coral *Montipora millepora* | Chan et al., 2018 | MG878677 | Chan et al., 2018 | MG878792 |
| CEL-JJ17-04 | *Cantellius arcuatus* | Jeju Island, Korea on coral *Montipora millepora* | Chan et al., 2018 | MG878678 | Chan et al., 2018 | MG878793 |
| CEL-JJ18-02 | *Cantellius arcuatus* | Jeju Island, Korea on coral *Montipora millepora* | Chan et al., 2018 | MG878679 | Chan et al., 2018 | MG878794 |
| CEL-JJ18-03 | *Cantellius arcuatus* | Jeju Island, Korea on coral *Montipora millepora* | Chan et al., 2018 | MG878680 | Chan et al., 2018 | MG878795 |
| CEL-JJ18-04 | *Cantellius arcuatus* | Jeju Island, Korea on coral *Montipora millepora* | Chan et al., 2018 | MG878681 | Chan et al., 2018 | MG878796 |
| CEL-JJ19-02 | *Cantellius arcuatus* | Big Rock,  Jeju Island, Korea on coral *Montipora millepora* | Chan et al., 2018 | MG878682 | Chan et al., 2018 | MG878797 |
| CEL-JJ19-03 | *Cantellius arcuatus* | Big Rock,  Jeju Island, Korea on coral *Montipora millepora* | Chan et al., 2018 | MG878683 | Chan et al., 2018 | MG878798 |
| CEL-JJ19-04 | *Cantellius arcuatus* | Big Rock,  Jeju Island, Korea on coral *Montipora millepora* | Chan et al., 2018 | MG878684 | Chan et al., 2018 | MG878799 |
| CEL-JJ20-02 | *Cantellius arcuatus* | Jeju Island, Korea on coral *Montipora millepora* | Chan et al., 2018 | MG878685 | Chan et al., 2018 | MG878800 |
| CEL-JJ20-03 | *Cantellius arcuatus* | Jeju Island, Korea on coral *Montipora millepora* | Chan et al., 2018 | MG878686 | Chan et al., 2018 | MG878801 |
| CEL-JJ20-04 | *Cantellius arcuatus* | Jeju Island, Korea on coral *Montipora millepora* | Chan et al., 2018 | MG878687 | Chan et al., 2018 | MG878802 |
| CEL-JJ23-02 | *Cantellius arcuatus* | Jeju Island, Korea on coral *Montipora millepora* | Chan et al., 2018 | MG878688 | Chan et al., 2018 | MG878806 |
| CEL-JJ23-03 | *Cantellius arcuatus* | Jeju Island, Korea on coral *Montipora millepora* | Chan et al., 2018 | MG878689 | Chan et al., 2018 | MG878807 |
| CEL-JJ23-04 | *Cantellius arcuatus* | Jeju Island, Korea on coral *Montipora millepora* | Chan et al., 2018 | MG878690 | Chan et al., 2018 | MG878808 |
| CEL-JJ25-02 | *Cantellius arcuatus* | Jeju Island, Korea on coral *Montipora millepora* | Chan et al., 2018 | MG878691 | Chan et al., 2018 | MG878809 |
| CEL-JJ25-03 | *Cantellius arcuatus* | Jeju Island, Korea on coral *Montipora millepora* | Chan et al., 2018 | MG878692 | Chan et al., 2018 | MG878810 |
| CEL-JJ25-05 | *Cantellius arcuatus* | Jeju Island, Korea on coral *Montipora millepora* | Chan et al., 2018 | MG878693 | Chan et al., 2018 | MG878811 |
| CEL-JJ28-03 | *Cantellius arcuatus* | Jeju Island, Korea on coral *Montipora millepora* | Chan et al., 2018 | MG878694 | Chan et al., 2018 | MG878812 |
| CEL-JJ28-04 | *Cantellius arcuatus* | Jeju Island, Korea on coral *Montipora millepora* | Chan et al., 2018 | MG878695 | Chan et al., 2018 | MG878813 |
| CEL-JJ28-05 | *Cantellius arcuatus* | Jeju Island, Korea on coral *Montipora millepora* | Chan et al., 2018 | MG878696 | Chan et al., 2018 | MG878814 |
| CEL-JJ33-02 | *Cantellius arcuatus* | Jeju Island, Korea on coral *Montipora millepora* | Chan et al., 2018 | MG878697 | Chan et al., 2018 | MG878815 |
| CEL-JJ33-03 | *Cantellius arcuatus* | Jeju Island, Korea on coral *Montipora millepora* | Chan et al., 2018 | MG878698 | Chan et al., 2018 | MG878816 |
| CEL-JJ33-04 | *Cantellius arcuatus* | Jeju Island, Korea on coral *Montipora millepora* | Chan et al., 2018 | MG878699 | Chan et al., 2018 | MG878817 |
| CEL-JJ34-02 | *Cantellius arcuatus* | Jeju Island, Korea on coral *Montipora millepora* | Chan et al., 2018 | MG878700 | Chan et al., 2018 | MG878818 |
| CEL-JJ34-03 | *Cantellius arcuatus* | Jeju Island, Korea on coral *Montipora millepora* | Chan et al., 2018 | MG878701 | Chan et al., 2018 | MG878819 |
| CEL-JJ34-04 | *Cantellius arcuatus* | Jeju Island, Korea on coral *Montipora millepora* | Chan et al., 2018 | MG878702 | Chan et al., 2018 | MG878820 |
| CEL-JJ35-01 | *Cantellius arcuatus* | Beom Sum, Jeju Island, Korea on coral *Montipora* | Chan et al., 2018 | MG878703 | Chan et al., 2018 | MG878821 |
| CEL-JJ36-02 | *Cantellius arcuatus* | Beom Sum,  Jeju Island, Korea on coral *Montipora millepora* | Chan et al., 2018 | MG878704 | Chan et al., 2018 | MG878822 |
| CEL-JJ36-03 | *Cantellius arcuatus* | Beom Sum, Jeju Island, Korea on coral *Montipora millepora* | Chan et al., 2018 | MG878705 | Chan et al., 2018 | MG878823 |
| CEL-JJ36-04 | *Cantellius arcuatus* | Beom Sum,  Jeju Island, Korea on coral *Montipora millepora* | Chan et al., 2018 | MG878706 | Chan et al., 2018 | MG878824 |
| CEL-JJ38-02 | *Cantellius arcuatus* | Beom Sum,  Jeju Island, Korea on coral *Montipora millepora* | Chan et al., 2018 | MG878716 | Chan et al., 2018 | MG878828 |
| CEL-JJ38-03 | *Cantellius arcuatus* | Beom Sum, Jeju Island, Korea on coral *Montipora millepora* | Chan et al., 2018 | MG878717 | Chan et al., 2018 | MG878829 |
| CEL-JJ38-04 | *Cantellius arcuatus* | Beom Sum, Jeju Island, Korea on coral *Montipora millepora* | Chan et al., 2018 | MG878718 | Chan et al., 2018 | MG878830 |
| CEL-JJ41-03 | *Cantellius arcuatus* | Beom Sum,  Jeju Island, Korea on coral *Montipora millepora* | Chan et al., 2018 | MG878719 | Chan et al., 2018 | MG878836 |
| CEL-JJ41-04 | *Cantellius arcuatus* | Beom Sum, Jeju Island, Korea on coral *Montipora millepora* | Chan et al., 2018 | MG878720 | Chan et al., 2018 | MG878837 |
| CEL-JJ41-05 | *Cantellius arcuatus* | Beom Sum, Jeju Island, Korea on coral *Montipora millepora* | Chan et al., 2018 | MG878721 | Chan et al., 2018 | MG878838 |
| CEL-JJ43-02 | *Cantellius arcuatus* | Beom Sum, Jeju Island, Korea on coral *Montipora millepora* | Chan et al., 2018 | MG878722 | Chan et al., 2018 | MG878842 |
| CEL-JJ43-03 | *Cantellius arcuatus* | Beom Sum, Jeju Island, Korea on coral *Montipora millepora* | Chan et al., 2018 | MG878723 | Chan et al., 2018 | MG878843 |
| CEL-JJ43-04 | *Cantellius arcuatus* | Beom Sum, Jeju Island, Korea on coral *Montipora millepora* | Chan et al., 2018 | MG878724 | Chan et al., 2018 | MG878844 |
| KC-104-02 | *Cantellius arcuatus* | Nishidomara, Kochi, Japan on coral *Pavona decussata* | Chan et al., 2018 | MG878725 | Chan et al., 2018 | MG878848 |
| KC-104-03 | *Cantellius arcuatus* | Nishidomara, Kochi, Japan on coral *Pavona decussata* | Chan et al., 2018 | MG878726 | Chan et al., 2018 | MG878849 |
| KC-107-02 | *Cantellius arcuatus* | Nishidomara, Kochi, Japan on coral *Dipsastraea lizardensis* | Chan et al., 2018 | MG878727 | Chan et al., 2018 | MG878850 |
| KC-107-03 | *Cantellius arcuatus* | Nishidomara, Kochi, Japan on coral *Dipsastraea lizardensis* | Chan et al., 2018 | MG878728 | Chan et al., 2018 | MG878851 |
| KC-107-04 | *Cantellius arcuatus* | Nishidomara, Kochi, Japan on coral *Dipsastraea lizardensis* | Chan et al., 2018 | MG878729 | Chan et al., 2018 | MG878852 |
| KC-115-01 | *Cantellius arcuatus* | Nishidomara, Kochi, Japan on coral *Coscinaraea columna* | Chan et al., 2018 | MG878730 | Chan et al., 2018 | MG878853 |
| KC-115-02 | *Cantellius arcuatus* | Nishidomara, Kochi, Japan on coral *Coscinaraea columna* | Chan et al., 2018 | MG878731 | Chan et al., 2018 | MG878854 |
| KC-115-03 | *Cantellius arcuatus* | Nishidomara, Kochi, Japan on coral *Coscinaraea columna* | Chan et al., 2018 | MG878732 | Chan et al., 2018 | MG878855 |
| KC-119-09 | *Cantellius arcuatus* | Nishidomara, Kochi, Japan on coral *Plesiastrea versipora* | Chan et al., 2018 | MG878733 | Chan et al., 2018 | MG878856 |
| MY-17-02 | *Cantellius arcuatus* | Tinggi Island, Malaysia on coral *Montipora undata* | Chan et al., 2018 | MG878734 | Chan et al., 2018 | MG878857 |
| MY-17-03 | *Cantellius arcuatus* | Tinggi Island, Malaysia on coral *Montipora undata* | Chan et al., 2018 | MG878735 | Chan et al., 2018 | MG878858 |
| MY-83-01 | *Cantellius arcuatus* | Tinggi Island, Malaysia on coral *Montipora undata* | Chan et al., 2018 | MG878736 | Chan et al., 2018 | MG878859 |
| MY-83-02 | *Cantellius arcuatus* | Tinggi Island, Malaysia on coral *Montipora undata* | Chan et al., 2018 | MG878737 | Chan et al., 2018 | MG878860 |
| MY-83-03 | *Cantellius arcuatus* | Tinggi Island, Malaysia on coral *Montipora undata* | Chan et al., 2018 | MG878738 | Chan et al., 2018 | MG878861 |
| PNG-43-01 | *Cantellius arcuatus* | Madang, Papua New Guinea on coral *Psammocora contigua* | Chan et al., 2018 | MG878739 | Chan et al., 2018 | MG878862 |
| PNG-43-04 | *Cantellius arcuatus* | Madang, Papua New Guinea on coral *Psammocora contigua* | Chan et al., 2018 | MG878740 | Chan et al., 2018 | MG878863 |
| PNG-43-06 | *Cantellius arcuatus* | Madang, Papua New Guinea on coral *Psammocora contigua* | Chan et al., 2018 | MG878741 | Chan et al., 2018 | MG878864 |
| PNG-43-07 | *Cantellius arcuatus* | Madang, Papua New Guinea on coral *Psammocora contigua* | Chan et al., 2018 | MG878742 | Chan et al., 2018 | MG878865 |
| brev1 | *Cantellius brevitergum* | *Acropora* sp. | — | — | Zweifler et al., 2020 | MG840383 |
| CEL-KT14-1 | *Cantellius*  *euspinulosum* | Nanwan, Kenting, Taiwan on coral *Porites* sp. | — | — | Tsang et al., 2014 | KF776145 |
| CEL-KT14-4 | *Cantellius*  *euspinulosum* | Nanwan, Kenting, Taiwan on coral *Porites* sp. | — | — | Tsang et al., 2014 | KF776146 |
| CEL-KT16-1 | *Cantellius*  *euspinulosum* | Green Island, Taiwan on coral *Acropora lutkeni* | Chan et al., 2018 | MG878750 | Tsang et al., 2014 | KF776147 |
| CEL-KT27-3 | *Cantellius*  *euspinulosum* | Nanwan, Kenting, Taiwan on coral *Porites* sp. | — | — | Tsang et al., 2014 | KF776148 |
| CEL- JJ08-02 | *Cantellius* cf. *euspinulosum* | Youngsuri,  Jeju Island, Korea on coral *Alveopora japonica* | Chan et al., 2018 | MG878707 | Chan et al., 2018 | MG878770 |
| CEL- JJ08-03 | *Cantellius* cf. *euspinulosum* | Youngsuri,  Jeju Island, Korea on coral *Alveopora japonica* | Chan et al., 2018 | MG878708 | Chan et al., 2018 | MG878771 |
| CEL- JJ08-04 | *Cantellius* cf. *euspinulosum* | Youngsuri,  Jeju Island, Korea on coral *Alveopora japonica* | Chan et al., 2018 | MG878709 | Chan et al., 2018 | MG878772 |
| CEL- JJ37-02 | *Cantellius* cf. *euspinulosum* | Beom Sum, Jeju Island, Korea on coral *Alveopora japonica* | Chan et al., 2018 | MG878710 | Chan et al., 2018 | MG878825 |
| CEL- JJ37-03 | *Cantellius* cf. *euspinulosum* | Beom Sum, Jeju Island, Korea on coral *Alveopora japonica* | Chan et al., 2018 | MG878711 | Chan et al., 2018 | MG878826 |
| CEL- JJ37-04 | *Cantellius* cf. *euspinulosum* | Beom Sum, Jeju Island, Korea on coral *Alveopora japonica* | Chan et al., 2018 | MG878712 | Chan et al., 2018 | MG878827 |
| CEL- JJ39-01 | *Cantellius* cf. *euspinulosum* | Beom Sum, Jeju Island, Korea on coral *Alveopora japonica* | Chan et al., 2018 | MG878713 | Chan et al., 2018 | MG878831 |
| CEL- JJ39-02 | *Cantellius* cf. *euspinulosum* | Beom Sum, Jeju Island, Korea on coral *Alveopora japonica* | Chan et al., 2018 | MG878714 | Chan et al., 2018 | MG878832 |
| CEL- JJ39-03 | *Cantellius* cf. *euspinulosum* | Beom Sum, Jeju Island, Korea on coral *Alveopora japonica* | Chan et al., 2018 | MG878715 | Chan et al., 2018 | MG878833 |
| ASIZCR000203 | *Cantellius hoegi* | Dong-Qing-Wan, Lanyu Island, Taiwan on coral *Pachyseris speciosa* | Achituv et al.,2009 | FJ379314 | Achituv et al.,2009 | FJ379302 |
| ASIZCR000204 | *Cantellius hoegi* | Dong-Qing-Wan, Lanyu Island, Taiwan on coral *Pachyseris speciosa* | Achituv et al.,2009 | FJ379315 | Achituv et al.,2009 | FJ379303 |
| ASIZCR000205 | *Cantellius hoegi* | Dong-Qing-Wan, Lanyu Island, Taiwan on coral *Pachyseris speciosa* | Achituv et al.,2009 | FJ379316 | Achituv et al.,2009 | FJ379304 |
| iwa1 | *Cantellius iwayama* | *Pachyseirs* sp. | Achituv et al. (Unpublished) | MG944315 | Zweifler et al., 2020 | MG840381 |
| CEL-GI170-1 | *Cantellius pallidus* | General Rock, Green Island, Taiwan on coral *Pocillopora* sp. | Chan et al., 2018 | MG878746 | Tsang et al., 2014 | KF776151 |
| CEL-SU3-1 | *Cantellius pallidus* | Neipei Sea, Suao, Taiwan on coral *Pocillopora damicornis* | — | — | Tsang et al., 2014 | KF776152 |
| AS_BKKC_Capal | *Cantellius pallidus* | Kenting, Taiwan on coral *Pocillopora damicornis* | Achituv et al.,2009 | FJ379317 | Achituv et al.,2009 | AM497881 |
| sec1 | *Cantellius secundus* | Unknown | — | — | Zweifler et al., 2020 | MG840385 |
| ASIZCR000206 | *Cantellius sextus* | Kenting, Taiwan on coral *Pachyseris* sp. | Achituv et al.,2009 | FJ379311 | Achituv et al.,2009 | FJ379299 |
| ASIZCR000207 | *Cantellius sextus* | Kenting, Taiwan on coral *Pachyseris* sp. | Achituv et al.,2009 | FJ379312 | Achituv et al.,2009 | FJ379300 |
| ASIZCR000208 | *Cantellius sextus* | Kenting, Taiwan on coral *Pachyseris* sp. | Achituv et al.,2009 | FJ379313 | Achituv et al.,2009 | FJ379301 |
| sept1 | *Cantellius septimus* | *Montipora* sp. | — | — | Zweifler et al., 2020 | MG840386 |
| sept2 | *Cantellius septimus* | *Montipora* sp. | — | — | Zweifler et al., 2020 | MG840387 |
| sept3 | *Cantellius septimus* | *Montipora* sp. | — | — | Zweifler et al., 2020 | MG840388 |
| FR_1 | *Cantellius* cf. *sumbawae* | *Distichopora* sp. | — | — | Zweifler et al., 2020 | MG840372 |
| FR_2 | *Cantellius* cf. *sumbawae* | *Distichopora* sp. | — | — | Zweifler et al., 2020 | MG840373 |
| FR_3 | *Cantellius* cf. *sumbawae* | *Distichopora* sp. | — | — | Zweifler et al., 2020 | MG840374 |
| FR_4 | *Cantellius* cf. *sumbawae* | *Distichopora* sp. | — | — | Zweifler et al., 2020 | MG840375 |
| FR_5 | *Cantellius* cf. *sumbawae* | *Distichopora* sp. | — | — | Zweifler et al., 2020 | MG840376 |
| FR_6 | *Cantellius* cf. *sumbawae* | *Distichopora* sp. | — | — | Zweifler et al., 2020 | MG840377 |
| FR_7 | *Cantellius* cf. *sumbawae* | *Distichopora* sp. | — | — | Zweifler et al., 2020 | MG840378 |
| CEL-SU46-1 | *Cantellius transversalis* | Suao, Taiwan on coral  *Acropora elseyi* | — | — | Tsang et al., 2014 | KF776154 |
| BIU-FR | *Cantellius transversalis* | — | Achituv et al. (Unpublished) | MG893082 | — | — |
| trans1 | *Cantellius transversalis* | — | — | — | Achituv et al. (Unpublished) | MG840389 |
| CEL-JJ03-02 | *Pyrgomina oulastreae* | Site 316,  Jeju Island, Korea on coral *Oulastrea crispate* | Chan et al., 2018 | MG878635 | Chan et al., 2018 | MG878758 |
| CEL-JJ03-03 | *Pyrgomina oulastreae* | Site 316,  Jeju Island, Korea on coral *Oulastrea crispate* | Chan et al., 2018 | MG878636 | Chan et al., 2018 | MG878759 |
| CEL-JJ03-04 | *Pyrgomina oulastreae* | Site 316,  Jeju Island, Korea on coral *Oulastrea crispate* | Chan et al., 2018 | MG878637 | Chan et al., 2018 | MG878760 |
| CEL-JJ04-02 | *Pyrgomina oulastreae* | Site 316,  Jeju Island, Korea on coral *Psammocora* spp. | Chan et al., 2018 | MG878638 | Chan et al., 2018 | MG878761 |
| CEL-JJ04-03 | *Pyrgomina oulastreae* | Site 316,  Jeju Island, Korea on coral *Psammocora* spp. | Chan et al., 2018 | MG878639 | Chan et al., 2018 | MG878762 |
| CEL-JJ04-04 | *Pyrgomina oulastreae* | Site 316,  Jeju Island, Korea on coral *Psammocora* spp. | Chan et al., 2018 | MG878640 | Chan et al., 2018 | MG878763 |
| CEL-JJ05-02 | *Pyrgomina oulastreae* | Site 316,  Jeju Island, Korea on coral *Oulastrea crispate* | Chan et al., 2018 | MG878641 | Chan et al., 2018 | MG878764 |
| CEL-JJ05-03 | *Pyrgomina oulastreae* | Site 316,  Jeju Island, Korea on coral *Oulastrea crispate* | Chan et al., 2018 | MG878642 | Chan et al., 2018 | MG878765 |
| CEL-JJ05-04 | *Pyrgomina oulastreae* | Site 316,  Jeju Island, Korea on coral *Oulastrea crispate* | Chan et al., 2018 | MG878643 | Chan et al., 2018 | MG878766 |
| CEL-JJ06-02 | *Pyrgomina oulastreae* | Site 316,  Jeju Island, Korea on coral *Oulastrea crispate* | Chan et al., 2018 | MG878644 | Chan et al., 2018 | MG878767 |
| CEL-JJ06-03 | *Pyrgomina oulastreae* | Site 316, Jeju Island, Korea on coral *Oulastrea crispate* | Chan et al., 2018 | MG878645 | Chan et al., 2018 | MG878768 |
| CEL-JJ06-04 | *Pyrgomina oulastreae* | Site 316,  Jeju Island, Korea on coral *Oulastrea crispate* | Chan et al., 2018 | MG878646 | Chan et al., 2018 | MG878769 |
| CEL-JJ10-02 | *Pyrgomina oulastreae* | Site 316,  Jeju Island, Korea on coral *Oulastrea crispate* | Chan et al., 2018 | MG878647 | Chan et al., 2018 | MG878773 |
| CEL-JJ10-03 | *Pyrgomina oulastreae* | Site 316,  Jeju Island, Korea on coral *Oulastrea crispate* | Chan et al., 2018 | MG878648 | Chan et al., 2018 | MG878774 |
| CEL-JJ10-04 | *Pyrgomina oulastreae* | Site 316,  Jeju Island, Korea on coral *Oulastrea crispate* | Chan et al., 2018 | MG878649 | Chan et al., 2018 | MG878775 |
| CEL-JJ11-02 | *Pyrgomina oulastreae* | Site 316,  Jeju Island, Korea on coral *Oulastrea crispate* | Chan et al., 2018 | MG878661 | Chan et al., 2018 | MG878776 |
| CEL-JJ11-03 | *Pyrgomina oulastreae* | Site 316,  Jeju Island, Korea on coral *Oulastrea crispate* | Chan et al., 2018 | MG878662 | Chan et al., 2018 | MG878777 |
| CEL-JJ11-04 | *Pyrgomina oulastreae* | Site 316,  Jeju Island, Korea on coral *Oulastrea crispate* | Chan et al., 2018 | MG878663 | Chan et al., 2018 | MG878778 |
| CEL-JJ21-02 | *Pyrgomina oulastreae* | Big Rock, 1^st^ Dive, Jeju Island, Korea on coral *Oulastrea crispate* | Chan et al., 2018 | MG878650 | Chan et al., 2018 | MG878803 |
| CEL-JJ21-03 | *Pyrgomina oulastreae* | Big Rock, 1^st^ Dive, Jeju Island, Korea on coral *Oulastrea crispate* | Chan et al., 2018 | MG878651 | Chan et al., 2018 | MG878804 |
| CEL-JJ21-04 | *Pyrgomina oulastreae* | Big Rock, 1^st^ Dive, Jeju Island, Korea on coral *Oulastrea crispate* | Chan et al., 2018 | MG878652 | Chan et al., 2018 | MG878805 |
| CEL-JJ40-01 | *Pyrgomina oulastreae* | Site 323,  Jeju Island, Korea on coral *Psammocora* spp. | Chan et al., 2018 | MG878653 | Chan et al., 2018 | MG878834 |
| CEL-JJ40-02 | *Pyrgomina oulastreae* | Site 323,  Jeju Island, Korea on coral *Psammocora* spp | Chan et al., 2018 | MG878654 | Chan et al., 2018 | MG878835 |
| CEL-JJ42-02 | *Pyrgomina oulastreae* | Beom Sum,  Jeju Island, Korea on coral *Oulastrea crispate* | Chan et al., 2018 | MG878655 | Chan et al., 2018 | MG878839 |
| CEL-JJ42-03 | *Pyrgomina oulastreae* | Beom Sum,  Jeju Island, Korea on coral *Oulastrea crispate* | Chan et al., 2018 | MG878656 | Chan et al., 2018 | MG878840 |
| CEL-JJ42-04 | *Pyrgomina oulastreae* | Beom Sum,  Jeju Island, Korea on coral *Oulastrea crispate* | Chan et al., 2018 | MG878657 | Chan et al., 2018 | MG878841 |
| CEL-JJ44-03 | *Pyrgomina oulastreae* | Site 324, Beom Sum, Jeju Island, Korea on coral *Oulastrea crispate* | Chan et al., 2018 | MG878658 | Chan et al., 2018 | MG878845 |
| CEL-JJ44-04 | *Pyrgomina oulastreae* | Site 324, Beom Sum, Jeju Island, Korea on coral *Oulastrea crispate* | Chan et al., 2018 | MG878659 | Chan et al., 2018 | MG878846 |
| CEL-JJ44-05 | *Pyrgomina oulastreae* | Site 324, Beom Sum, Jeju Island, Korea on coral *Oulastrea crispate* | Chan et al., 2018 | MG878660 | Chan et al., 2018 | MG878847 |
| CEL-KT78-4 | *Pyrgoma cancellatum* | Nanwan, Kenting, Taiwan on coral *Turbinaria frondens* | — | — | Tsang et al., 2014 | KF776167 |
| CEL-KT78-5 | *Pyrgoma cancellatum* | Nanwan, Kenting, Taiwan on coral *Turbinaria frondens* | — | — | Tsang et al., 2014 | KF776168 |
| 1-NSB(341) | *Pyrgoma cancellatum* | *Turbinarai* sp. | — | — | Simon-Blecher  et al., 2007 | AM497905 |
| UF9278 | *Pyrgoma* sp.1 | Philippines on coral *Tubastrea* sp. | Malay & Michonneau, 2014 | HG970566 | — | — |
| UF13133 | *Pyrgoma* sp.2 | Philippines on coral *Turbinaria* sp | Malay & Michonneau, 2014 | HG970567 | Malay & Michonneau, 2014 | HG970506 |
| ORI | *Pyrgoma* sp.2 | South Africa on coral *Turbinaria* sp | Malay & Michonneau, 2014 | HG970565 | Malay & Michonneau, 2014 | HG970505 |
| UF13131 | *Nobia orbicellae* | Philippines on coral  *Goniopora* sp. | Malay & Michonneau, 2014 | HG970536 | Malay & Michonneau, 2014 | HG970478 |
| UF26330 | *Adna anglica* | Spain on coral *Oculina patagonica* | Malay & Michonneau, 2014 | HG970570 | Malay & Michonneau, 2014 | HG970509 |
| UF26338 | *Adna anglica* | Spain on coral *Oculina patagonica* | Malay & Michonneau, 2014 | HG970569 | Malay & Michonneau, 2014 | HG970508 |
| UF26329 | *Adna anglica* | Spain on coral *Oculina patagonica* | Malay & Michonneau, 2014 | HG970568 | Malay & Michonneau, 2014 | HG970507 |
| TAU<ISR>: AR29618 | *Adna anglica* | — | Achituv et al. (Unpublished) | KU986739 | — | — |
