# Supplementary material for: DNA-based diversity assessment reveals a new coral barnacle, Cantellius alveoporae sp. nov. (Balanomorpha: Pyrgomatidae) exclusively associated with the high latitude coral Alveopora japonica in the waters of southern Korea
Source: PeerJ. 2021 Apr 29;9:e11284. doi: 10.7717/peerj.11284 (PMC8088765; doi:10.7717/peerj.11284)
Supplement: Table S2 [file peerj-09-11284-s002.docx]

**Table S2 Mean Kimura 2-Parameter distances for the concatenated dataset (COI + 12S) within and among species of *Cantellius*.**

|  | 1 | 2 | 3 | 4 | 5 | 6 | 7 | 8 | 9 | 10 | 11 | 12 | 13 | 14 | 15 | 16 | 17 | 18 | 19 | 20 | 21 | 22 | 23 |
| --- | --- | --- | --- | --- | --- | --- | --- | --- | --- | --- | --- | --- | --- | --- | --- | --- | --- | --- | --- | --- | --- | --- | --- |
| 1. *Cantellius* sp.1 | 0.014 |  |  |  |  |  |  |  |  |  |  |  |  |  |  |  |  |  |  |  |  |  |  |
| 2. *Cantellius arcuatus* | 0.082 | 0.002 |  |  |  |  |  |  |  |  |  |  |  |  |  |  |  |  |  |  |  |  |  |
| 3.*Cantellius euspinulosum* | 0.064 | 0.060 | 0.013 |  |  |  |  |  |  |  |  |  |  |  |  |  |  |  |  |  |  |  |  |
| 4. *Cantellius arcuatum* | 0.063 | 0.061 | 0.018 | 0.004 |  |  |  |  |  |  |  |  |  |  |  |  |  |  |  |  |  |  |  |
| 5. *Cantellius septimus* | 0.051 | 0.046 | 0.034 | 0.037 | 0.005 |  |  |  |  |  |  |  |  |  |  |  |  |  |  |  |  |  |  |
| 6.*Cantellius transversalis* | 0.073 | 0.062 | 0.048 | 0.048 | 0.033 | 0.006 |  |  |  |  |  |  |  |  |  |  |  |  |  |  |  |  |  |
| 7. *Cantellius acutum* | 0.082 | 0.075 | 0.058 | 0.058 | 0.056 | 0.070 | 0.022 |  |  |  |  |  |  |  |  |  |  |  |  |  |  |  |  |
| 8. *Cantellius* sp.7 | 0.095 | 0.100 | 0.067 | 0.064 | 0.062 | 0.078 | 0.068 | n/a |  |  |  |  |  |  |  |  |  |  |  |  |  |  |  |
| 9. *Cantellius brevitergum* | 0.083 | 0.065 | 0.058 | 0.068 | 0.055 | 0.069 | 0.074 | 0.078 | n/a |  |  |  |  |  |  |  |  |  |  |  |  |  |  |
| 10. *Cantellius alveoporae*sp. nov | 0.083 | 0.069 | 0.064 | 0.064 | 0.022 | 0.067 | 0.079 | 0.092 | 0.053 | 0.002 |  |  |  |  |  |  |  |  |  |  |  |  |  |
| 11. *Cantellius hoegi* | 0.025 | 0.086 | 0.070 | 0.067 | 0.058 | 0.090 | 0.087 | 0.102 | 0.086 | 0.089 | 0.010 |  |  |  |  |  |  |  |  |  |  |  |  |
| 12. *Cantellius iwayama* | 0.030 | 0.072 | 0.060 | 0.059 | 0.054 | 0.071 | 0.088 | 0.100 | 0.080 | 0.077 | 0.029 | n/a |  |  |  |  |  |  |  |  |  |  |  |
| 13. *Cantellius pallidus* | 0.088 | 0.088 | 0.061 | 0.061 | 0.039 | 0.066 | 0.083 | 0.090 | 0.069 | 0.075 | 0.090 | 0.083 | 0.005 |  |  |  |  |  |  |  |  |  |  |
| 14. *Cantellius secundus* | 0.081 | 0.067 | 0.044 | 0.051 | 0.058 | 0.055 | 0.050 | 0.045 | 0.089 | 0.062 | 0.089 | 0.086 | 0.072 | n/a |  |  |  |  |  |  |  |  |  |
| 15. *Cantellius sextus* | 0.090 | 0.066 | 0.044 | 0.043 | 0.050 | 0.067 | 0.072 | 0.100 | 0.059 | 0.069 | 0.093 | 0.087 | 0.065 | 0.048 | 0.008 |  |  |  |  |  |  |  |  |
| 16. *Cantellius* cf. *sumbawae* | 0.053 | 0.054 | 0.045 | 0.047 | 0.043 | 0.048 | 0.066 | 0.064 | 0.069 | 0.051 | 0.054 | 0.063 | 0.054 | 0.064 | 0.046 | 0.003 |  |  |  |  |  |  |  |
| 17. *Pyrgomina oulastreae* | 0.121 | 0.142 | 0.127 | 0.131 | 0.114 | 0.139 | 0.135 | 0.157 | 0.109 | 0.141 | 0.125 | 0.122 | 0.146 | 0.132 | 0.147 | 0.109 | 0.002 |  |  |  |  |  |  |
| 18.*Pyrgoma cancellatum* | 0.129 | 0.170 | 0.142 | 0.131 | 0.095 | 0.138 | 0.140 | 0.122 | 0.120 | 0.158 | 0.118 | 0.121 | 0.172 | 0.148 | 0.116 | 0.114 | 0.150 | 0.000 |  |  |  |  |  |
| 19. *Pyrgoma*sp.1 | 0.137 | 0.128 | 0.147 | 0.154 | 0.137 | 0.147 | 0.146 | 0.154 | n/a | 0.145 | 0.142 | 0.122 | 0.190 | n/a | 0.141 | n/a | 0.140 | n/a | n/a |  |  |  |  |
| 20.*Pyrgoma*  sp.2 (from PHL) | 0.134 | 0.147 | 0.132 | 0.137 | 0.136 | 0.148 | 0.143 | 0.166 | 0.137 | 0.151 | 0.137 | 0.141 | 0.153 | 0.177 | 0.139 | 0.142 | 0.160 | 0.056 | 0.141 | n/a |  |  |  |
| 21.*Pyrgoma*  sp.2 (from SA) | 0.128 | 0.144 | 0.115 | 0.119 | 0.112 | 0.132 | 0.136 | 0.158 | 0.126 | 0.137 | 0.129 | 0.129 | 0.141 | 0.158 | 0.131 | 0.122 | 0.145 | 0.021 | 0.125 | 0.066 | n/a |  |  |
| 22. *Nobia orbicellae* | 0.144 | 0.155 | 0.147 | 0.142 | 0.147 | 0.154 | 0.148 | 0.159 | 0.155 | 0.162 | 0.146 | 0.144 | 0.156 | 0.151 | 0.162 | 0.152 | 0.152 | 0.151 | 0.160 | 0.162 | 0.154 | n/a |  |
| 23. *Adna anglica* | 0.123 | 0.147 | 0.122 | 0.127 | 0.118 | 0.132 | 0.131 | 0.153 | 0.119 | 0.139 | 0.128 | 0.121 | 0.133 | 0.132 | 0.147 | 0.115 | 0.036 | 0.123 | 0.149 | 0.163 | 0.147 | 0.155 | 0.005 |

n/a: not available
